# Editorial Notes and Comments

**Published:** 1900-04

**Authors:** 


### *EDITORIAL NOTES AND COMMENTS.*

The Business office of THE JOURNAL-RECORD is 305, 306 Fitten Building.

The Editorial office is 318-319-320 The Prudential.

Address all Business communications to Dr. M. B. Hutchins, Mgr.

Make remittances payable to THE ATLANTA JOURNAL-RECORD OF MEDICINE.

On matters pertaining to the Editorial and Original Communications address Dr. Bernard Wolff, Atlanta.

Reprints of original articles will be furnished at cost price. Requests for the same should always be made *on the manuscript.*

We will present, post-paid, on request, to each contributor of an original article, twenty (20) marked copies of THE JOURNAL-RECORD containing such article.

THE American public appears to suffer from a periodic spasm of virtue. Like other convulsive seizures, it leaves no trace, but establishes a tendency to recurrence. The occasion of the present attack is a foreign actress of shady reputation and a nasty play.

The anomaly of the situation consists in the fact that an immoral play can be cast from a book which for fifteen years has basked in the reputation among those who do not affiliate with the class of the truly good, as inculcating high moral principles, and therefore especially adapted to the needs of incautious youth. It is said to teach with solemn warning the inevitable retribution that overtakes those who tread the primrose path of dalliance, and is therefore a finger-post to guide to the straight and narrow way. What strange transformation has occurred by which the chaste and virtuous book has become the licentious and suggestive play? Has the playwright served the novelist a shabby trick and picked out for emphasis all the unshadowed grossness, leaving unnoticed the tender moral tones and vitiated the purpose of the book? Is it not rather that the *mise en scène* has been changed and incorruptible has thus put on corruption? That which is said in secret may not be cried from the housetops; that which is told in Gath may not be published in Askelon. The difference between the play and the book is rather apparent than real. It depends upon the psychological attitude, the angle of mental vision of the observer.

We inherit our conception of morality from the English; we borrow our plays from the French. If the two do not harmonize, are we justified in the employment of the Procrustean method of adjusting the one to the other?

We must either test the ductility of our moral conceptions to the point of general inclusiveness, or retain the rigid puritanic severity that rejects everything incommensurable with it. There is no middle ground except the offensive pharisaic self-consciousness, that says with broadened phylacteries, "I am holier than thou."

Let us attempt to be consistent upon nice questions involving a distinction between real and human nature itself, and its depiction and delineation. If we cannot be consistent, let us be as consistent as we can. The book, the play, the statue, the painting, are Art (note the capital), and therefore pure, or they are all base, prurient, tarred with the same stick according to the personal equation of the observer. It is the latter that must decide; the higher court of a man's own conscience, that which resides *in immo pectore*.

But why select Daudet's Sapho as an especial object of attack by those whose sense of propriety has been suddenly, outrageously shocked. Is "the moral lesson" it is supposed to contain less apparent in its attractively fleshly garnishment than in many books which have lain for years free from censorial molestation and purchased *viva voce* in the book shops? Is Sapho more injurious to public morals than Bourget's Mensonges, Balzac's Contes Drolatiques, Guy de Maupassant's Une Vie, Rousseau's Confessions, D'Annunzio's Triumph of Death, Tolstoi's Anna Karénina and the

Kreutzer Sonata, all of which are translated into English and readily purchasable?

To make an olla podrida of the good, bad and worse, all are immoral, libels on human nature and corrupting. They are not read for moral lessons (the Sunday-school library can supply these), but as foul things that appeal to a foul pruriency, as the smell of offal attracts the jackal.

If the moral lesson contained in Sapho is the object of quest and the sole attraction of the book, by all means let us have an expurgated edition. It would be an interesting test of the book's popularity to say the least, and of its moral educational value. It might be expedient at the same time to substitute a title not quite so suggestive of sexual perversion.

Mark Twain said that if the phrase "and it came to pass" were taken out of the Book of Mormon, it would shrink to the size of a pamphlet. If Daudet's book were stripped of immorality as one strips the coats of an onion (a just because malodorous simile), the moral lesson would approximate in size the core of the onion, something not every one may find. Yet Sapho is not a whit worse than its class. It has attained a temporary notoriety and the present process of suppression is very much like removing one fly from the apothecary's ointment and leaving many overlooked because submerged.

If the public wants moral lessons, is imperiously clamoring for them, in the name of decency, go to the Bible and the church for them and not the brothel and the latrine. W.

---

A CORRESPONDENT has sent us a communication from the alleged St. Luke's Hospital, Niles, Michigan. The singular methods pursued by this institution have been exposed by the Journal of the American Medical Association, and the Cleveland

Journal of Medicine, and do not require here any further comment or publicity. The letter is printed in typewriter style and is of the nature of a circular. The Medical Staff is exceedingly imposing; the literary and technical degrees of its members fearful and wonderful, and the institutions from which they graduated of the most formidable importance. This much shows on the face, but the fact that the St. Luke's Hospital of Niles, Michigan, is a crass and cheap fake, is only apparent to the initiated. The available school of suckers in the West having been exhausted, the attention of "the workers" seems now to have turned southward for a further supply. The letter is as follows, and reminds one of the experience of Jacky Horner with the Christmas pie:

NILES, MICHIGAN, February 24th, 1900.

DEAR DOCTOR:—In looking over our record book, we find that we have no member from your locality on our Medical Staff, and from a list of names handed us we have selected you (subject to your confirmation) as a good physician to represent our interests in your city. The plan of organization of our hospital is entirely different to that of any other hospital, being financially beneficial to the physician sending us cases as well as ourselves. We have already a number of physicians from your State on our medical staff, and trust that we may have the pleasure of adding your name to this list.

We will pay you a commission of 25% of the operation fee of all surgical cases, and 10% of the fee for all medical cases you may send to our hospital for treatment. We charge nothing for nursing patients day or night, as that part of the expense is taken from our "Nursing Fund." We do charge, however, for board and room, ranging from $1.50 to $2.00 per day, according to the location selected by the patient.

Should you wish to consult us at any time regarding difficult cases we will freely give you what assistance and advice we can, and will make microscopical analysis of specimens sent us free of charge.

It is our invariable rule never to appoint more than one physician in each locality, and should he so desire it, after accepting the appointment and paying the membership fee, and will send us a list of names, not exceeding twelve,—including the local newspapers,—we will write individual letters recommending him to them, copy of which is herewith enclosed.

To each of our members we issue a very neat and attractive certificate setting forth that he is a member in good standing on our medical staff and entitled to all the advantages and privileges of our hospital. We also issue in addition to our certificate a neat pocket lithographed membership ticket, which we believe, if rightly displayed, will be the means of securing many patients for our hospital. We charge as a membership fee according to the grade of certificate selected (see physicians' application blank), which goes to make up our "Nursing Fund."

Trusting that you will fill out the enclosed application form and return it to us with a remittance covering the amount of the priced certificate you may select, we remain, Fraternally, yours,

A. C. PROBERT, M.D., President.
32° K. of P.

I see you are Medical Examiner for the K. of P. Shake! as I am a member of this noble order.

Just received a letter from Dr. H. D. Hattery, Physician and Pharmacist of Logansport, Indiana, who writes us: "The certificate on sheepskin to hand for which please accept my thanks. It more than meets with all my expectations." (Signed) H. D. HATTERY, M.D.

Dr. Hattery is a graduate of Miami Medical College of Cincinnati, Ohio, class of 1874. DR. PROBERT.

---

IN the *Medico-Legal Journal* for December, there appears a very interesting article by Dr. H. G. Garrigues, read before the Section in Medicine at the New York Academy of Medicine, on the subject of Premature Burial. This subject, of vital interest to every one, has not met the due consideration which it deserves. There are a sufficient number of authentic cases of premature burials to warrant us in considering such in the light of a possibility. Some of the deductions and arguments of Dr. Garrigues are not without force. He says:

"I am far from agreeing either in one extreme or the other, but I do believe that premature burials are not very rare, and I base this belief on the fact that graves so extremely rarely are reopened, and still it is asserted that on such occasions sometimes it becomes evident that the inmate of the grave has revived in his coffin.

Secondly, and this consideration has more weight with me than the first, I base my belief in the comparative frequency of premature burials on the numerous cases in which people have had narrow escapes from being buried alive.

Thirdly, I base my belief on the unreliability of the so-called signs of death, with the sole exception of unquestionable putrefaction of vital organs.

Fourthly, I base it on the absence of proper laws to protect the apparently dead against live burial.

Fifthly, and lastly, I base it on the carelessness with which death certificates are signed by physicians."

The writer further adds which is most truthful in character, "As a matter of fact, many physicians do not even glance at the supposed bodies of their patients, but sign the certificates of death on the report of friends or strangers present at the supposed death. What then is needed to put an end to this disgraceful remnant of barbarism?"

The writer then proposes the following remedy:

First. Only authorized practitioners of medicine should decide, whether a person is dead or not.

Secondly. The blanks for certificates of death should contain questions in regard to the chief signs of death, and the physician signing the certificate should answer each question with "yes" or "no," besides declaring that he personally has examined the body.

Thirdly. It should be made a crime to do anything to the supposed dead that would cause pain or injury to a living person, before the certificate is signed.

As long as nothing of the kind is done, none of us has any guarantee that he will not be buried alive, thrown into a glowing furnace, or be killed by the performers of autopsies, the undertakers with their ice-box, or the embalmers with their solution of arsenic.

---

WE note in a pamphlet recently received extolling the great value of Dr. Geo. Leininger's Formaldehyd Inhaler, an advertisement on the last page of the Chicago School of Clinical Medicine. A great many physicians in the South know nothing of this clinical school, but if they are to form an opinion of it in conjunction with this rankest of quack advertisements which daily fill our secular papers, then its standard will certainly be placed on a very low basis. A testimonial is also given by one of its professors which still further degrades this institution in the eyes of reputable physicians.

